# Antioxidant and anti-inflammatory effects of allicin in the kidney of an experimental model of metabolic syndrome

**DOI:** 10.7717/peerj.16132

**Published:** 2023-09-27

**Authors:** Abraham Said Arellano Buendia, Juan Gabriel Juárez Rojas, Fernando García-Arroyo, Omar Emiliano Aparicio Trejo, Fausto Sánchez-Muñoz, Raúl Argüello-García, Laura Gabriela Sánchez-Lozada, Rafael Bojalil, Horacio Osorio-Alonso

**Affiliations:** 1 Doctorado en Ciencias Biológicas y de la Salud, Universidad Autónoma Metropolitana, Mexico, Xochimilco, Mexico; 2 Fisiopatología Cardio-Renal, Instituto Nacional de Cardiología Ignacio Chávez, Mexico, Tlalpan, México; 3 Endocrinología, Instituto Nacional de Cardiología Ignacio Chávez, Mexico, Tlalpan, México; 4 Inmunología, Instituto Nacional de Cardiología Ignacio Chávez, Mexico, Tlalpan, México; 5 Genética y Biología Molecular, Centro de Investigación y de Estudios Avanzados del IPN, México, Gustavo A. Madero, México; 6 Atención a la Salud, Universidad Autónoma Metropolitana, Mexico, Xochimilco, México

**Keywords:** Metabolic syndrome, Allicin, Chronic kidney disease, Inflammation, Oxidative stress, Hypertension

## Abstract

**Background:**

Recent studies have suggested that metabolic syndrome (MS) encompasses a group of risk factors for developing chronic kidney disease (CKD). This work aimed to evaluate the antioxidant and anti-inflammatory effects of allicin in the kidney from an experimental model of MS.

**Methods:**

Male Wistar rats (220–250 g) were used, and three experimental groups (*n* = 6) were formed: control (C), metabolic syndrome (MS), and MS treated with allicin (16 mg/Kg/day, gastric gavage) (MS+A). MS was considered when an increase of 20% in at least three parameters (body weight, systolic blood pressure (SBP), fasting blood glucose (FBG), or dyslipidemia) was observed compared to the C group. After the MS diagnosis, allicin was administered for 30 days.

**Results:**

Before the treatment with allicin, the MS group showed more significant body weight gain, increased SBP, and FBG, glucose intolerance, and dyslipidemia. In addition, increased markers of kidney damage in urine and blood. Moreover, the MS increased oxidative stress and inflammation in the kidney compared to group C. The allicin treatment prevented further weight gain, reduced SBP, FBG, glucose intolerance, and dyslipidemia. Also, markers of kidney damage in urine and blood were decreased. Further, the oxidative stress and inflammation were decreased in the renal cortex of the MS+A compared to the MS group.

**Conclusion:**

Allicin exerts its beneficial effects on the metabolic syndrome by considerably reducing systemic and renal inflammation as well as the oxidative stress. These effects were mediated through the Nrf2 pathway. The results suggest allicin may be a therapeutic alternative for treating kidney injury induced by the metabolic syndrome risk factors.

## Introduction

Metabolic syndrome (MS) is defined as a series of disorders considered risk factors for developing cardiovascular diseases (CVD) and diabetes (Wang et al., 2020). These factors include abdominal obesity, hyperglycemia, insulin resistance, hypertension, and dyslipidemia (Wang et al., 2020; Dommermuth & Ewing, 2018; Sperling et al., 2015). MS represents one of the major public health problems of the 21^st^ century worldwide, with a prevalence of approximately 25% in American adults. The prevalence has increased not only in the United States and Europe but also in some countries belonging to the Asian continent.

Some reports indicate that each one of the risk factors associated to MS can lead to developing chronic kidney disease (CKD); however, the more components there are, the greater the risk of suffering from CKD (Huh et al., 2017; Kawamoto et al., 2019; Wu et al., 2021; Alizadeh et al., 2018). The risk of CKD is 1.34 times higher in patients with MS than in healthy patients (Li et al., 2022). The relationship between MS and the incidence of CKD involves mechanisms such as inflammation, oxidative stress (OS), insulin resistance, renin-angiotensin-aldosterone system (RAAS) activation, endothelial damage, alterations in carbohydrate and lipid metabolism, as well as ectopic accumulation of lipids in the kidney (Lin et al., 2022; García-Arroyo et al., 2021; Zhang & Lerman, 2017).

In humans, lipid peroxidation correlated directly to body mass index (BMI) and waist circumference (Zhang & Lerman, 2017). In addition, hypercholesterolemia increases the production of oxygen free radicals leading to the oxidation of low-density lipoprotein cholesterol (LDL-c). Oxidized LDL (OxLDL-c) promotes the differentiation of monocytes to macrophages, which could directly damage vascular endothelium and is associated with microalbuminuria and alterations in estimated glomerular filtration rate (eGFR) (Lin et al., 2022; Zhang & Lerman, 2017; Sun et al., 2020).

On the other hand, adiposity leads to the production of proinflammatory cytokines such as interleukin-6 (IL-6), interleukin 1-β (IL-1β), and tumor necrosis factor-alpha (TNF-α), which are closely related to renal cell dysfunction and insulin resistance (Markova et al., 2019). Insulin resistance induces sodium retention, vasoconstriction and activation of the RAAS. Such alterations induce glomerular hyperfiltration, hyperperfusion glomerular, hypertrophy and segmental sclerosis, ultimately leading to chronic disease (Lin et al., 2022).

Due to easy access, low cost, cultural acceptability, fewer side effects, and the fact that good safety and efficacy have been reported, the use of medicinal plants is playing a critical role as a therapeutic option throughout the world (Anaeigoudari, Safari & Khazdair, 2021). Additionally, traditional medicine has recently gained acceptability because several plants have numerous beneficial effects (Yuan et al., 2016).

In this context, garlic (*Allium sativum*) is a spice rich in sulfur compounds, several of which have shown favorable effects in various diseases, including cardiovascular (Anaeigoudari, Safari & Khazdair, 2021; Sánchez-Gloria et al., 2022; Sharma & Rani, 2023). Diallyl thiosulfinate (allicin) is a sulfur compound in garlic, which is the main component formed when garlic is macerated or crushed (Sánchez-Gloria et al., 2022). Recently, several studies have shown that allicin has multiple beneficial effects, including antioxidant, immunomodulatory, antidiabetic, antihypertensive, cardioprotective, and nephroprotective (El-Sheakh et al., 2016; García-Trejo et al., 2017; Liu et al., 2010; Arellano-Buendía et al., 2018, 2020; Sánchez-Gloria et al., 2021, 2022). Therefore, considering allicin regulates several pathogenic mechanisms in cardiovascular diseases, the present study aimed to evaluate allicin’s antioxidant and anti-inflammatory effects on kidney damage induced by experimental MS.

## Materials and Methods

### Reagents

Sodium cholate hydrate, cholesterol, D-(+)-glucose, D-(−)-fructose, 2,4-dinitrophenylhydrazine (DNPH), and 4-hydroxynonenal (4HNE) were purchased from Sigma (St. Louis, MO, USA). Antibodies to nephrin (sc-376522), NGAL (sc-515876), IL-1β (sc-52012), IL-6 (sc-57315), TNF-α (sc-52746), and actin (sc-8432) were acquired from Santa Cruz Biotechnology, Inc., (Dallas, TX, USA). KIM-1 (GTX85067), Nrf2 (GTX103322), Keap1 (GTX60660), NFκB p65 (GTX102090), IκB (GTX82797), and PCNA (GTX100539) were from GeneTex (Irvine, CA, USA). Goat anti-rabbit and goat anti-mouse IgG-HRP were purchased from Cell Signaling Technology (Danvers, MA, USA). All other chemicals used were of the highest purity available.

### Experimental design

A total of 18 male Wistar rats (three rats per cage) weighing 200–220 g (6 weeks old) provided by the Instituto Nacional de Cardiología Ignacio Chávez vivarium were used. The rats were kept under controlled conditions with artificial light/dark cycles of 12 h and a temperature of 22 ± 24 °C. The rats were randomly divided into two groups. Control group (C) (*n* = 6 (Labdiet 5001 Rodent Diet™ maintenance diet)) and metabolic syndrome (MS) (*n* = 12 (Paigen-type diet + 11% sweetened beverage (3.8% glucose, 7.2% fructose) ad libitum)) (García-Arroyo et al., 2021; Hidalgo et al., 2020).

The two experimental groups were maintained for 30 days. MS was considered when an increase of 20% compared to C was observed in at least three of the following parameters: body weight, systolic blood pressure (SBP), fasting blood glucose (FBG), glucose intolerance, and dyslipidemia. The animals that did not meet these criteria were excluded. After MS diagnosis (30 days), this group was randomly subdivided into two subgroups: MS and MS-treated with allicin (16 mg/Kg/day, gastric gavage) (MS+A) group. Allicin was administered daily for 1 month. Synthetic allicin was resuspended in water (Argüello-García et al., 2010); a similar volume of purified water was administered to the control and MS group as a vehicle of the test compound. Blood and urine samples were collected on day 30 of follow-up and at the end of the experiment (day 60). After completion of the experimental protocols, the animals were sacrificed with sodium pentobarbital (65 mg/kg, i.p.) and exsanguination *via* the abdominal aorta and the kidneys were surgically removed. The kidneys were dissected into cortex and medulla. All tissue samples were snap frozen in liquid nitrogen and stored at −70 °C until further analysis.

### Ethics statement

All animal procedures were carried out following the guidelines for the care and use of laboratory animals established and published by the U.S. National Institutes of Health, the Mexican Federal Regulation for animal experimentation and care (NOM-062-ZOO-1999), and the disposal of biological residues (NOM-087-ECOL-1995), and approved by the Institutional Animal Care and Use Committee of the Instituto Nacional de Cardiología Ignacio Chávez (INC/CICUAL/001/2020, Institutional permit number 20-1144).

### Allicin synthesis

Allicin was synthesized as previously was described (Argüello-García et al., 2010). Briefly, 1 g of diallyl disulfide was dissolved in 5 ml of acetic acid with continuous stirring in an ice bath. Subsequently, hydrogen peroxide (1.5 mL, 30% v/v) was added and incubated for 30 min to continue the reaction. Next, the reaction was kept at 13 °C for 20 min, and placed on an ice bath for 5 h. Finally, the reaction was stopped by adding 15 ml of distilled water pH 6.5 and extracted with 30 ml of dichloromethane. Next, five extractions were made with 5% (p/v) Na2CO3 (20 mL each) and three extractions with distilled water (20 mL each). Finally, the solvent evaporated until a yellowish oil (allicin) remained. Allicin was resuspended in water at 2.5% (w/v) for stabilization and storage. The purity of the allicin obtained by this method is 90–92% (Argüello-García et al., 2010). Allicin was kept at −70 °C until it was used.

### Blood samples

Blood samples were obtained after overnight fasting. At each time point (30 and 60 days), 1.0 milliliter of blood was drawn from the tail vein and collected into heparinized tubes. Blood samples were centrifuged at 1,000×*g* for 15 min at 4 °C to obtain plasma and kept at −70 °C until used.

### Oral glucose tolerance test (OGTT)

The oral glucose tolerance test was performed on days 30 and 60 prior to sacrifice and after fasting for 12 h. Basal glucose concentration was measured using an Accu-Chek Active glucose meter (GC model), and a drop of blood was sampled from the tail vein. Subsequently, a glucose bolus (3 g/kg of body weight) was administered orally, and the blood glucose concentration was determined at 30, 60, 90, 120, 150, 180, and 210 min.

### Systolic blood pressure record (SBP)

SBP was recorded in rats at days 30 and 60 by a validated tail-cuff plethysmography method (Narco Biosystems, Austin, TX, USA).

### Plasma biochemistry

Triglycerides (TG), total cholesterol (TC), high-density lipoproteins cholesterol (HDL-c), low-density lipoprotein cholesterol (LDL-c), and creatinine levels were measured in plasma samples using commercial kits from Spinreact (Girona, Spain), following the manufacturer instructions (Triglycerides-LQ REF. 41030; Cholesterol-LQ REF. 41020; HDLc-D REF. 1001097; LDLc-D REF. 41023, and creatinine-J REF. 1001111). All parameters were read in a multimode reader (Sinergy H1; Biotek-Agilent, Santa Clara, CA, USA).

### Renal function

Urine samples were collected by placing the animals in metabolic cages (Nalgene, Rochester, NY, USA) for 24 h. The urine collected was centrifugated at 5,000×*g* for 15 min. The variables measured were diuresis, creatinine, proteinuria, microalbuminuria, and NGAL excretion.

At the end of the follow-up, the rats were euthanized with sodium pentobarbital (65 mg/kg, i.p.), and the kidneys were removed, split into cortex and medulla, snap frozen in liquid nitrogen, and stored at −70 °C until further analysis.

### Evaluation of oxidative stress

#### Determination of lipid peroxidation

Lipid peroxidation was assessed by measuring 4-Hydroxynonenal (4-HNE) using 1,1-3,3 tetramethoxypropane as standard. A total of 50 mg of tissue were homogenized in potassium phosphate buffer (20 mM) containing 0.5 M beta hydroxybutyrate (BHT) and protease inhibitor cocktail (Halt Protease Inhibitor; Thermo Scientific, Waltham, MA, USA). A solution of 1-methyl-2-phenylindole in a mixture of acetonitrile:methanol (3:1) was added to the kidney tissue samples and the reaction was started by adding 37% HCl. The absorbance was quantified at 586 nm in an ELISA plate reader. The results were expressed as nmol of 4-HNE/mg protein (Arellano-Buendía et al., 2018).

### Assay of oxidized protein

Carbonylated proteins were assessed as a marker of protein oxidation. In brief, the, samples (50 mg of tissue) were homogenized in potassium phosphate buffer (20 mM) with BHT (0.5 M) and protease inhibitor cocktail (Halt Protease Inhibitor; Thermo Scientific, Waltham, MA, USA) adjusted to pH 7.2 and incubated overnight with 10% streptomycin sulfate to remove nucleic acids. Subsequently, they were treated with dinitrophenylhydrazone (DNPH), HCl, and guanidine hydrochloride. Evaluation of carbonyl formation is made on the basis of protein hydrazone formation by reaction with DNPH. The absorbance was quantified at 370 nm in an ELISA plate reader. The content of carbonylated proteins was expressed as nanomoles of carbonyl/mg of protein (Arellano-Buendía et al., 2018).

### Mitochondrial respiratory complexes activities

As previously reported, mitochondrial complex activities were assessed in renal tissue homogenates (Aparicio-Trejo et al., 2017; Ortega-Domínguez et al., 2017). Briefly, complex I (CI) activity was measured based on their capacity to oxidize NADH while reducing DUB to DUbH2, which DCPIP then oxidizes. The CI activity is followed by the disappearance of oxidized DCPIP at 600 nm. CII activity was measured in a separate assay in the presence of 2.5 µM rotenone, using the succinate depend on CII capacity of reducing DUB, so the decrease in the absorbance at 600 nm is proportional to the activity of CII. Absorbance measurements were performed at 37 °C using a BioTek Cytation 7 microplate reader (Agilent Instruments, Santa Clara, CA, USA). The specific activity of each complex was determined by subtracting the activity in the presence of the appropriate inhibitor from the non-inhibited one. The results were expressed as nmol/min/mg protein determinated by the Bradford method.

### Activity of antioxidant enzymes in renal cortex

Tissue (100 mg) was homogenated in PBS buffer, and after centrifugation, the supernatant was used to determine the activity of antioxidant enzymes, according to the methods previously described (Briones-Herrera et al., 2018). Briefly, superoxide dismutase (SOD) activity was determined spectrophotometrically at 560 nm using NBT as the indicator reagen; the amount of protein that inhibited 50% of NBT was defined as one unit of SOD activity, and the results were expressed as U/mg protein of total protein. Glutathione peroxidase (GPx) activity was determined by measuring the disappearance of NADPH at 340 nm in a coupled reaction with GR and GPx. Units (U) were defined as the amount of enzyme that oxidizes one micromol NADPH/min, and data were expressed as U/mg total protein (Aparicio-Trejo et al., 2017). Absorbance measurements were performed at 37 °C using a BioTek Cytation 7 microplate reader (Agilent Instruments, Santa Clara, CA, USA).

### Cell fractionation

Fifty micrograms of tissue were homogenized in buffer-containing 3 mM imidazole and 250 mM sucrose. Homogenate was centrifuged at 3,400 rpm for 20 min at 4 °C. Pellet was homogenized in RIPA1X buffer for 2 h at 4 °C. Then the homogenate was centrifuged at 14,000 rpm for 5 min at 4 °C. The supernatant (cytoplasmic fraction) was lysed with RIPA 5X. The pellet (nuclear fraction) was lysed with RIPA1x buffer, frozen in liquid nitrogen, and stored at −70 °C until further analysis.

### Evaluation of markers of renal damage in urine

Urinary excretion of NGAL was quantified as a marker of renal damage. In brief, sample volumes corresponding to 15 μg of total protein were precipitated on ice for 30 min with 10% (w/v) trichloroacetic acid in PBS. Samples were centrifuged at 13,100×*g* for 10 min at 4 °C before washing pellets twice with ice-cold acetone. The samples were air-dried and dissolved in 50 mM phosphate buffer, pH 7.4, and stored at −70 °C until use.

### Expression of antioxidant proteins

Additionally, the protein expression of Nrf2 and Keap1 was assessed in the renal cortex homogenates, as described in the next section. The renal cortex was homogenized in 50 mM phosphate buffer, pH 7.4, kept cold on ice and supplemented with a protease inhibitor cocktail using a Potter Elvehjem homogenizer.

### Expression of inflammatory cytokines, renal damage markers and proteins related to oxidative stress by the western blot method

Total protein concentration in renal tissue homogenates, urine and serum were determined by the Bradford method using bovine serum albumin as standard. The samples were resuspended in Laemmli buffer (62.5 mmol/L Tris–HCl (pH 6.8), 10% (v/v) glycerol, 2% (w/v) SDS, 5% (w/v) 2-mercaptoethanol, and 0.05% (w/v) bromophenol blue). Equal protein amounts (7.5, 15 or 20 μg) were denatured in a gel loading buffer at 85 °C for 5 min, then loaded onto 10% SDS-polyacrylamide gels (SDS-PAGE), transferred to polyvinylidene difluoride (PVDF) membranes and incubated at 4 °C overnight with primary antibody diluted in Tris-buffered saline-Tween 0.1% solution. The protein bands were visualized with enhanced chemiluminescence reagents (Clarity Western ECL Substrate, Bio-Rad, Hercules, CA, USA) and their intensity was quantified using ImageJ (National Institutes of Health, Bethesda, MD, USA). The antibody dilutions were as follows: Nephrin (1:5,000), NGAL (1:20,000), IL-1β (1:5,000), IL-6 (1:5,000), TNF-α (1:5,000), actin (1:5,000), KIM-1 (1:5,000), Nrf2 (1:5,000), KEAP1 (1:5,000), NFκB p65 (1:5,000), IκB (1:5,000), and PCNA (1:2,000 dilution).

Positive immunoreactive bands were quantified and expressed as the ratio of the protein of interest test sample to loading control (actin) in arbitrary units (a.u.) as previously described (Arellano-Buendía et al., 2020).

### Statistical analysis

All data were expressed as means ± standard error of the mean (SEM). To analyze the 30-day data, the unpaired T test with Welch’s correction was used. To analyze the 60 day data, comparisons amongst groups were made using one-way analysis of variance followed by Tukey’s test using GraphPad version 9.0.1 (GraphPad Software, La Jolla, CA, USA). Differences were considered significant when *p* < 0.05.

## Results

### Validation of the model of metabolic syndrome and renal damage

**Figure 1:**
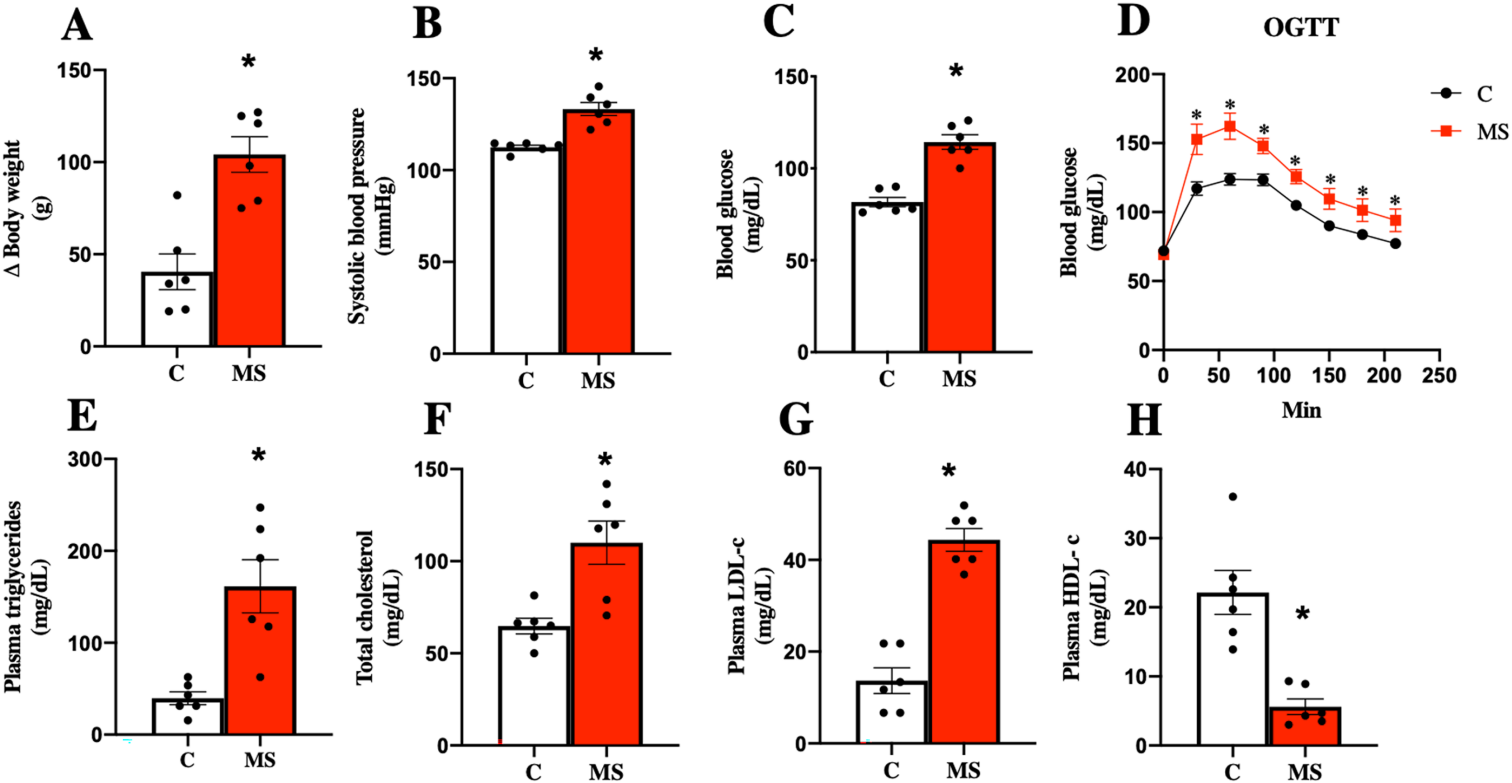
Validation of the metabolic syndrome model at 30 days. Body weight (A), systolic blood pressure (B), fasting blood glucose (C), and oral glucose tolerance test (OGTT) (D). Lipid profile in plasma: triglycerides (E), total cholesterol (F), LDL cholesterol (G), and HDL cholesterol (H). C, control; and MS, metabolic syndrome. Data are expressed as mean ± SEM, analysed by unpaired T test with Welch’s correction. Statistical significance was established as *p < 0.05 *vs.* C.

Considering the currently accepted clinical definition of MS in humans, an experimental model of metabolic syndrome was induced using a Paigen-type diet combined with a sugar-sweetened beverage as previously reported (García-Arroyo et al., 2021; Hidalgo et al., 2020). Figure 1 shows the results obtained after 30 days of dietary intervention. MS group gained more body weight gain and had, systolic blood pressure, and FBG (Figs. 1A–1C). On the other hand, the OGTT in MS indicated a greater intolerance to glucose uptake compared to the control group (Fig. 1D).

Dyslipidemia is another risk factor present in MS. The key features of dyslipidemia are high plasma TG, TC, LDL-c levels, and low HDL-c levels. We observed that TG, TC, and LDL-c levels increased in the MS group, while HDL-c was decreased when compared to the C group (Figs. 1E–1H).

**Figure 2:**
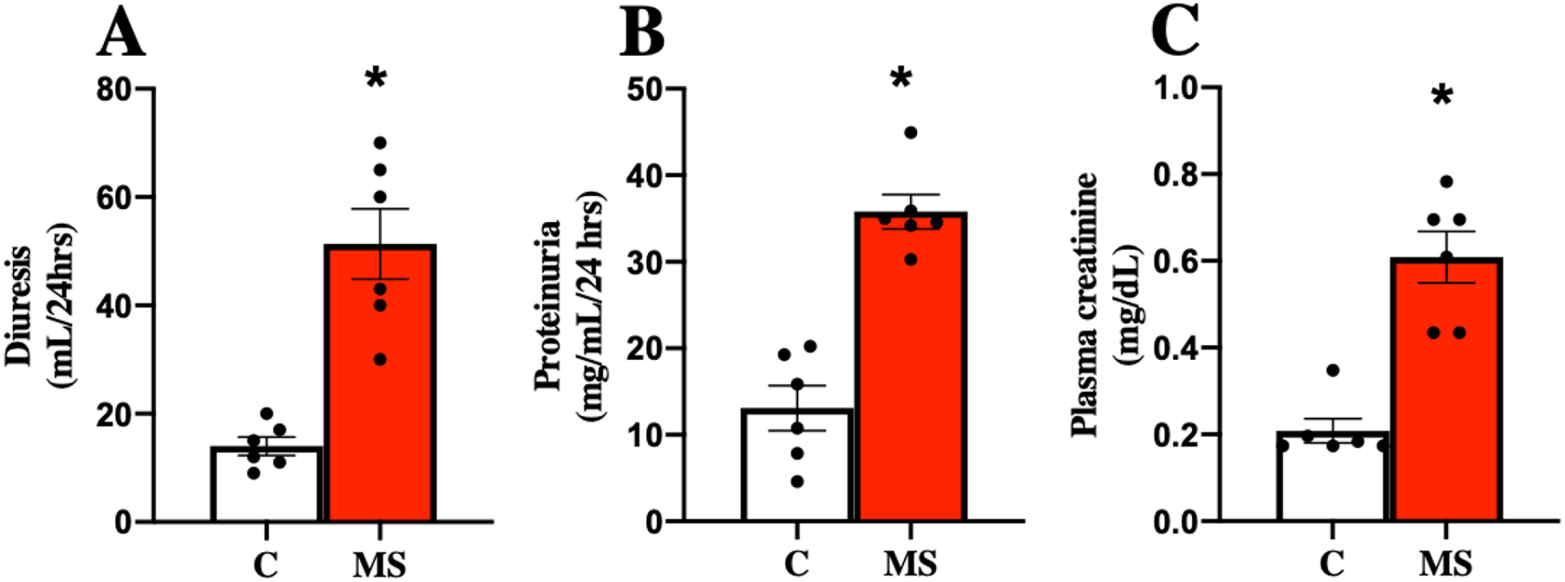
Kidney damage markers at 30 days. Diuresis (A), proteinuria (B), and plasma creatinine (C), respectively. C, control; and MS, metabolic syndrome. Data are expressed as mean ± SEM, analysed by unpaired T test with Welch’s correction. Statistical significance was established as *p < 0.05 *vs.* C.

MS has been closely related to CKD, so kidney damage markers in urine and serum were evaluated at 30 days of follow-up. The MS group presented increased urine volume, proteinuria, and plasma creatinine compared to the C group (Figs. 2A–2C).

Oxidative stress derived from mitochondrial dysfunction plays a key role in the development and progression of renal damage and is closely related to inflammation. Thus, we assessed the activity of mitochondrial complexes (I and II) as indirect indicators of renal oxidative stress in metabolic syndrome.

The mitochondria represent the major energy source in healthy conditions, but also it is also one of the kidney’s ROS producer sites. ROS production in the mitochondria is mainly due to dysfunction in the flow of the electron transfer system (complexes I and II). Thus, we evaluated the activities of mitochondrial CI and CII in the kidney. In the MS group, we observed a significant decrease in the activity of both complexes compared to the C group after 30 days (Figs. S1A and S1B), which suggests an impairment in the flow of electrons favoring ROS formation. The data suggest an increase in oxidative stress in the kidney during the MS.

In summary, after 30 days of follow-up, the experimental model of MS was validated and kidney damage and oxidative stress were demonstrated, so we next evaluated the effect of allicin.

### Allicin and its beneficial effects on the components of the metabolic syndrome

**Figure 3:**
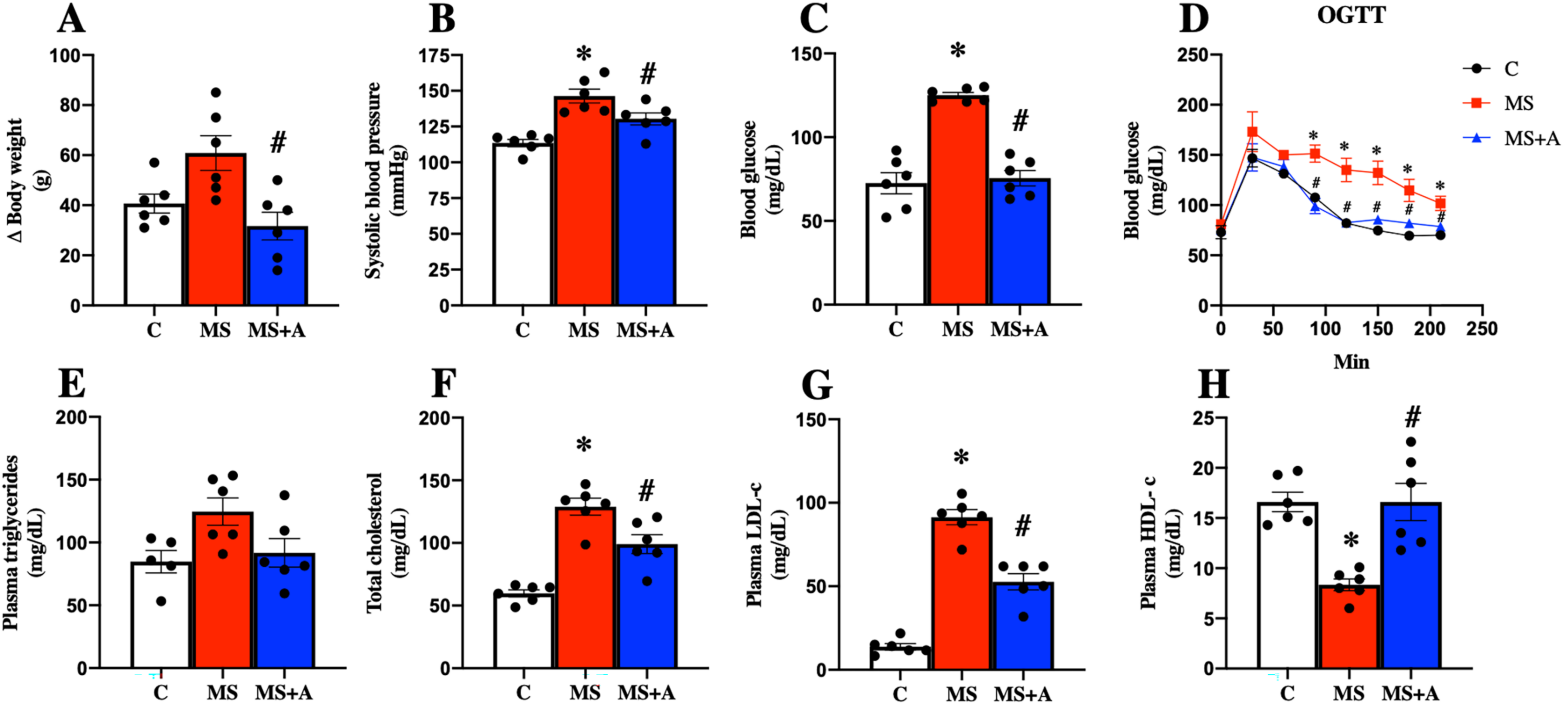
Effects of allicin at 60 days of follow-up on: Body weight (A), systolic blood pressure (B), fasting blood glucose (C), and oral glucose tolerance test (OGTT) (D). Lipid profile in plasma: triglycerides (E), total cholesterol (F), LDL cholesterol (G), and HDL cholesterol (H). C, control; MS, metabolic syndrome; MS+A, metabolic syndrome + allicin. Data are expressed as mean ± SEM and analysed by one-way ANOVA. Statistical significance was established as *p < 0.05 *vs.* C; ^#^*p* < 0.05 *vs.* MS.

After 60 days, the untreated MS group presented more significant body weight gain than the C group; interestingly, allicin treatment for 30 days fully reverted the weight gain. In addition, the MS group presented an increase in systolic blood pressure, and this effect was attenuated with the allicin administration, so the treatment exerted an antihypertensive effect. The FBG in the group of MS was increased compared to the C group but was reduced with the allicin treatment. This data suggest that the MS group induced intolerance to glucose uptake compared to the C group, and this was normalized by allicin treatment (Figs. 3A–3D).

Regarding the lipid profile, the MS group showed increased plasma levels of TC, LDL-c, and a decrease in HDL-c compared to the C group. The treatment with allicin provided beneficial effects since partially decreased plasma TC, LDL-c, and normalized plasma levels of HDL-c. On the other hand, when we evaluated plasma TG concentrations, we observed a trend to increase in the MS group and a return to control levels after allicin treatment (Figs. 3E–3H).

### Nephroprotective effects of allicin

**Figure 4:**
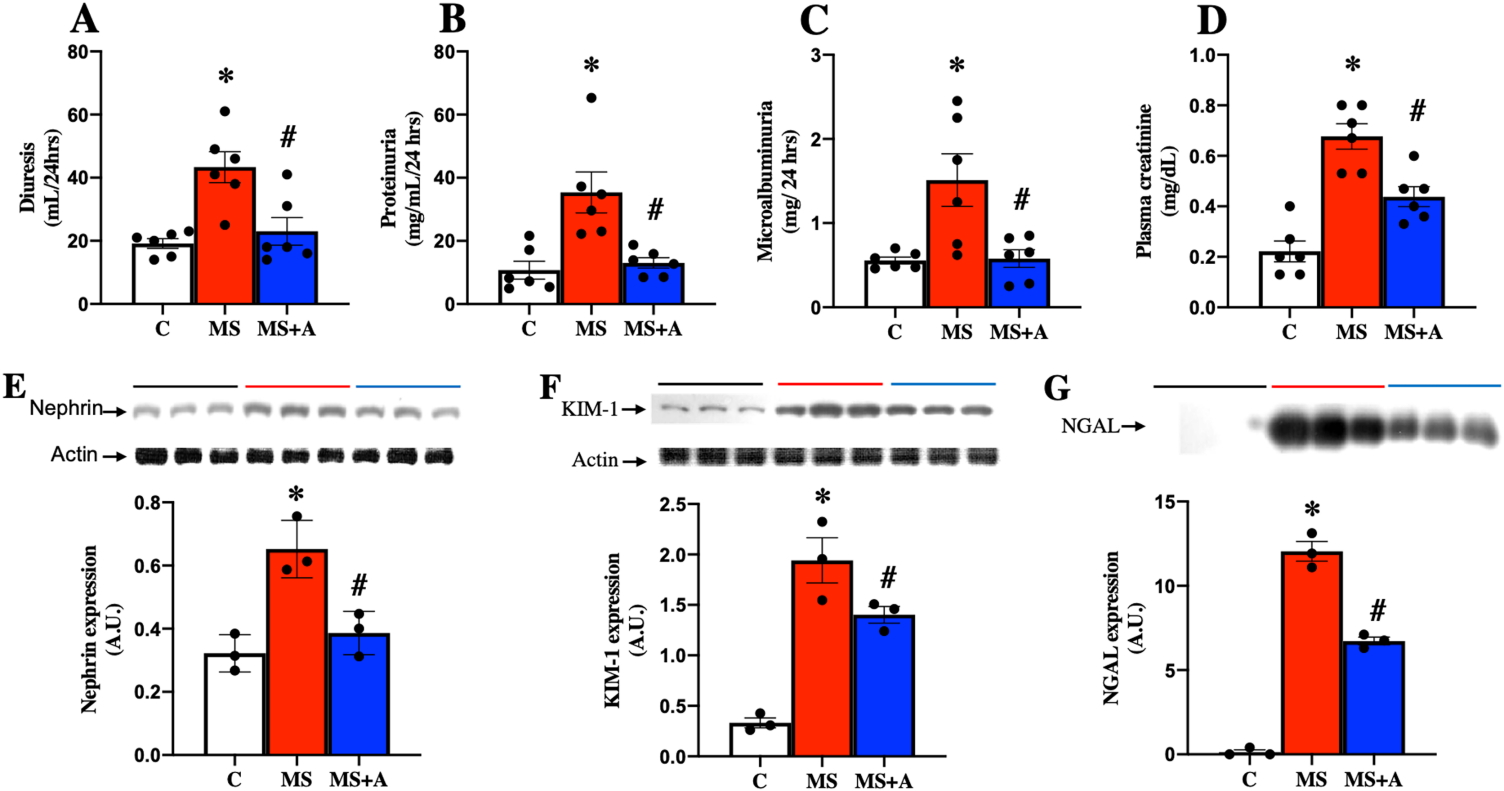
Effect of allicin at 60 days of follow-up on: Kidney damage markers. Diuresis (A), proteinuria (B), microalbuminuria (C), and plasma creatinine (D). In kidney tissue; Nephrin expression (E) and KIM-1 expression (F). In urine; NGAL expression (G). For representative Western blotting, 3 randomly selected samples per group were analysed respectively. C, control; MS, metabolic syndrome; and MS+A, metabolic syndrome + allicin. Data are expressed as mean ± SEM and analysed by one-way ANOVA. Statistical significance was established as *p < 0.005 *vs.* C; ^#^*p* < 0.05 *vs.* MS.

To evaluate the effects of allicin on kidney damage caused by MS, we assessed the following biochemical parameters: in plasma, the creatinine levels; in urine diuresis, proteinuria, and microalbuminuria. Figure 4 shows that in the MS group, the values of diuresis, proteinuria, microalbuminuria, and creatinine were increased significantly compared to the C group. The allicin treatment reverted proteinuria and microalbuminuria while partially reversed the increase in serum creatinine. These findings suggest that renal function wass impaired during metabolic syndrome and was improved with allicin (Figs. 4A–4D).

### Biomarkers of kidney damage in tissue: expression of nephrin, KIM-1, and NGAL

Nephrin and KIM-1 expressions in the renal cortex, were evaluated as markers at glomerular and tubular damage. The results showed a significantly higher protein expressions of nephrin and KIM-1 in the MS group than in the C group, suggesting renal damage at glomerular and tubular levels. However, treatment with allicin significantly decreased the expressions of nephrin and KIM-1. We also evaluated the urinary excretion of NGAL, another marker of tubular damage, which significantly increased in the MS group compared to the C group; however, allicin decreased the urinary excretion of this biomarker. Therefore the data suggest that allicin has a nephroprotective effect (Figs. 4E–4G).

Once we observed the nephroprotective effect of allicin, the next step was to evaluate the possible mechanisms by which allicin exerts its beneficial effects.

### Effects of allicin on oxidative stress in renal cortex

To evaluate the oxidative stress as a pathogenic mechanism in kidney damage, we evaluated the oxidation of lipids and proteins, the activity of mitochondrial complexes, the protein expression of Nrf2-Keap1, and the antioxidant activity of SOD and GPx.

**Figure 5:**
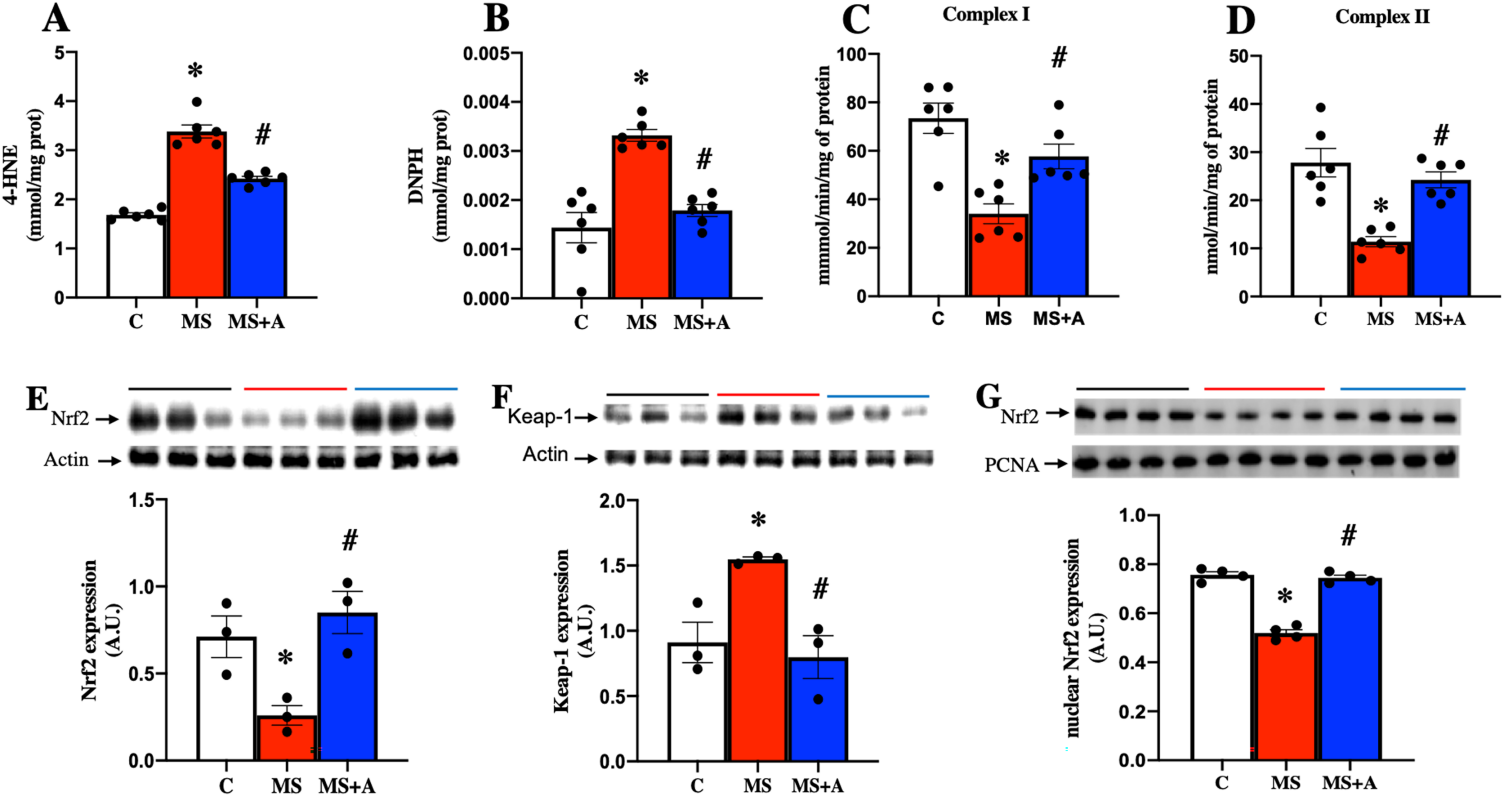
Effect of allicin on oxidative stress markers in the kidney at 60 days of follow-up. Lipid oxidation (A) and protein oxidation (B). Activity of mitochondrial complexes I (C) and II (D). Effects of allicin on expressions of total Nrf2 (E) and Keap-1 (F), and Nrf2 expression in nuclear extracts (G) of kidney cortex. For representative Western blotting, three randomly selected samples per group were analysed respectively. C, control; MS, metabolic syndrome; and MS+A, metabolic syndrome + allicin. Data are expressed as mean ± SEM and analysed by one-way ANOVA. Statistical significance was established as *p < 0.05 *vs.* C; ^#^*p* < 0.05 *vs.* MS.

In the MS group, a significant increase in the content of 4-HNE and DNPH as indicators of lipid and protein oxidation in the kidney were observed compared to the C group. The results confirm an increase in oxidative stress induced by MS. In contrast, allicin treatment decreased considerably the content of 4-HNE and DNPH compared with the MS group (Figs. 5A and 5B). Our findings show that allicin alleviated oxidative stress in the renal cortex.

### Effects of allicin on the mitochondrial activity of complexes (I and II) in kidney

After 60 days, MS rats presented a significant decrease in the mitochondrial activities of complexes I and II. On the other hand, allicin treatment significantly prevented the decrease in CI and CII activities induced by MS (Figs. 5C and 5D). These results suggest an increase in ROS formation in MS due to the impairment of electron flow in the mitochondria, and allicin protects the kidney through the improvement in the mitochondrial activities of complexes (I and II).

### Effects of allicin on Nrf2/Keap antioxidant pathway

We next analyzed the expression of Nrf2 and Keap1 in the renal cortex. This pathway is a master regulator of cellular antioxidant protection. The MS group presented lower expression of Nrf2 and increased expression of Keap1 compared to the C group. On the other hand, allicin treatment increased Nrf2 and decreased Keap1 expressions compared to the C group (Figs. 5E and 5F). It is well-known that Nrf2 promotes the expression of endogenous antioxidant enzymes when it translocates into the nucleus; thus, we also evaluated the nuclear expression of Nrf2. The MS group showed lower nuclear expression of Nrf2 compared to the C group; this was reverted with the allicin treatment (Fig. 5G). Later, the activity of endogenous antioxidant enzymes SOD and GPx was assessed as an indirect indicator of Nrf2 promoter activity. Interestingly, the evaluation of the activity of antioxidant enzymes showed that allicin treatment reversed the decrements in the activities of SOD and GPx observed in the MS group (Figs. S2A and S2B). These results suggest that allicin through the modulation of the antioxidant enzymes by Nrf2-Keap1 signal pathway reduced oxidative stress in kidney tissue induced by MS.

### Effects of allicin on inflammation markers in serum and renal cortex

**Figure 6:**
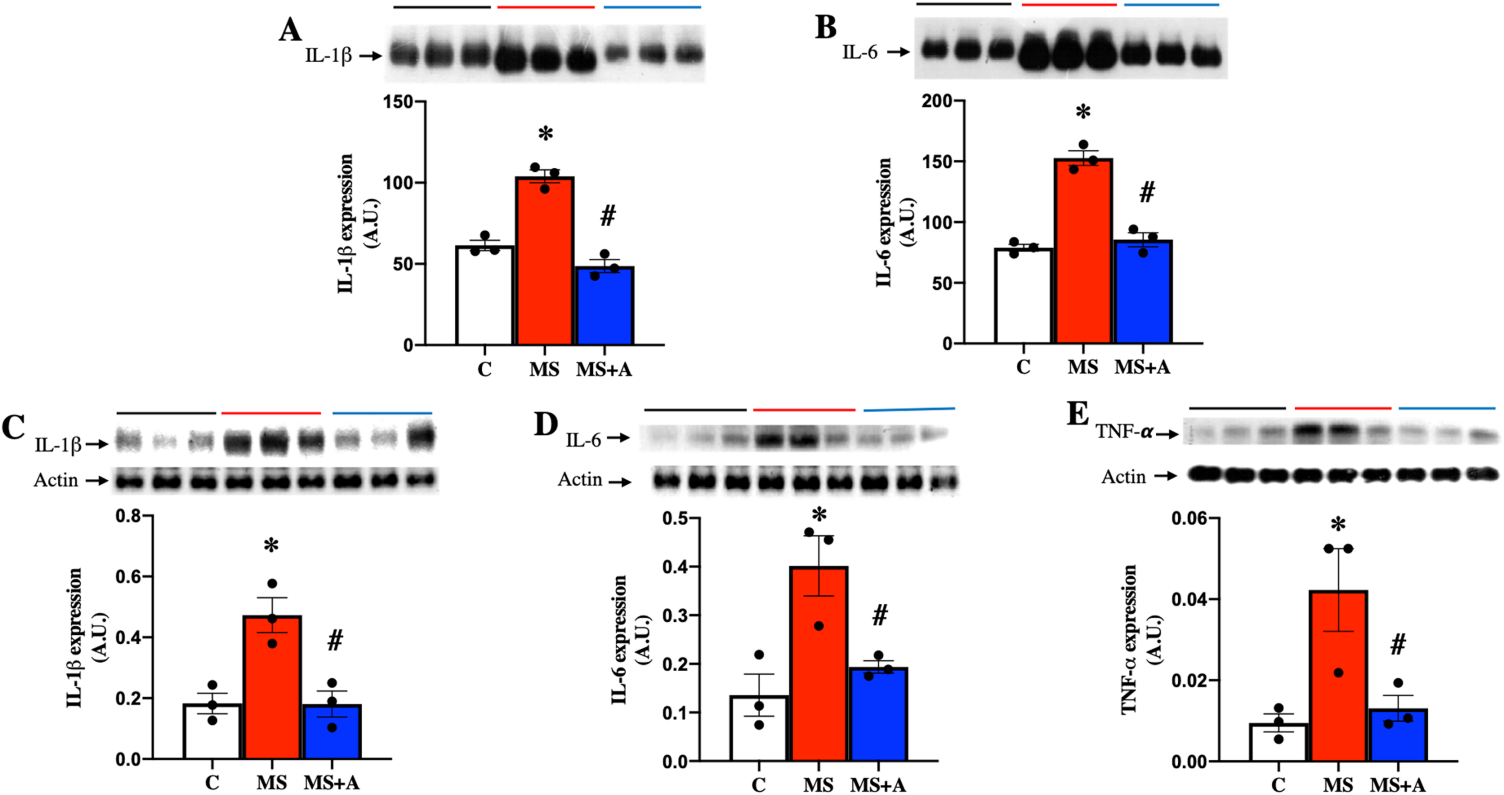
Effects of allicin on proinflammatory cytokines in plasma and kidney cortex at 60 days of follow-up. Expressions of interleukin 1 beta (IL-1β) (A) and interleukin 6 (IL-6) (B) in plasma. Expression of IL-1β (C), IL-6 (D), and TNF-α (E) in kidney cortex. For representative Western blotting, three randomly selected samples per group were analysed respectively. C, control; MS, metabolic syndrome; MS+A. Data are expressed as mean ± SEM and analysed by one-way ANOVA. Statistical significance was established as *p < 0.05 *vs.* C; ^#^*p* < 0.05 *vs.* MS.

To evaluate the effect of allicin on the proinflammatory cytokines, we analyzed the expressions of IL-1β, IL-6 and TNF-α in plasma and renal cortex. MS group showed a significant increase in the expression of IL-1β and IL-6 in plasma compared to group C, and allicin treatment significantly decreased their overexpression (Figs. 6A and 6B).

In the renal cortex the expressions of IL-1β, IL-6, and TNF-α also were significantly increased in the MS group compared to group C and treatment with allicin decreased the overexpression of those cytokines (Figs. 6C–6E).

**Figure 7:**
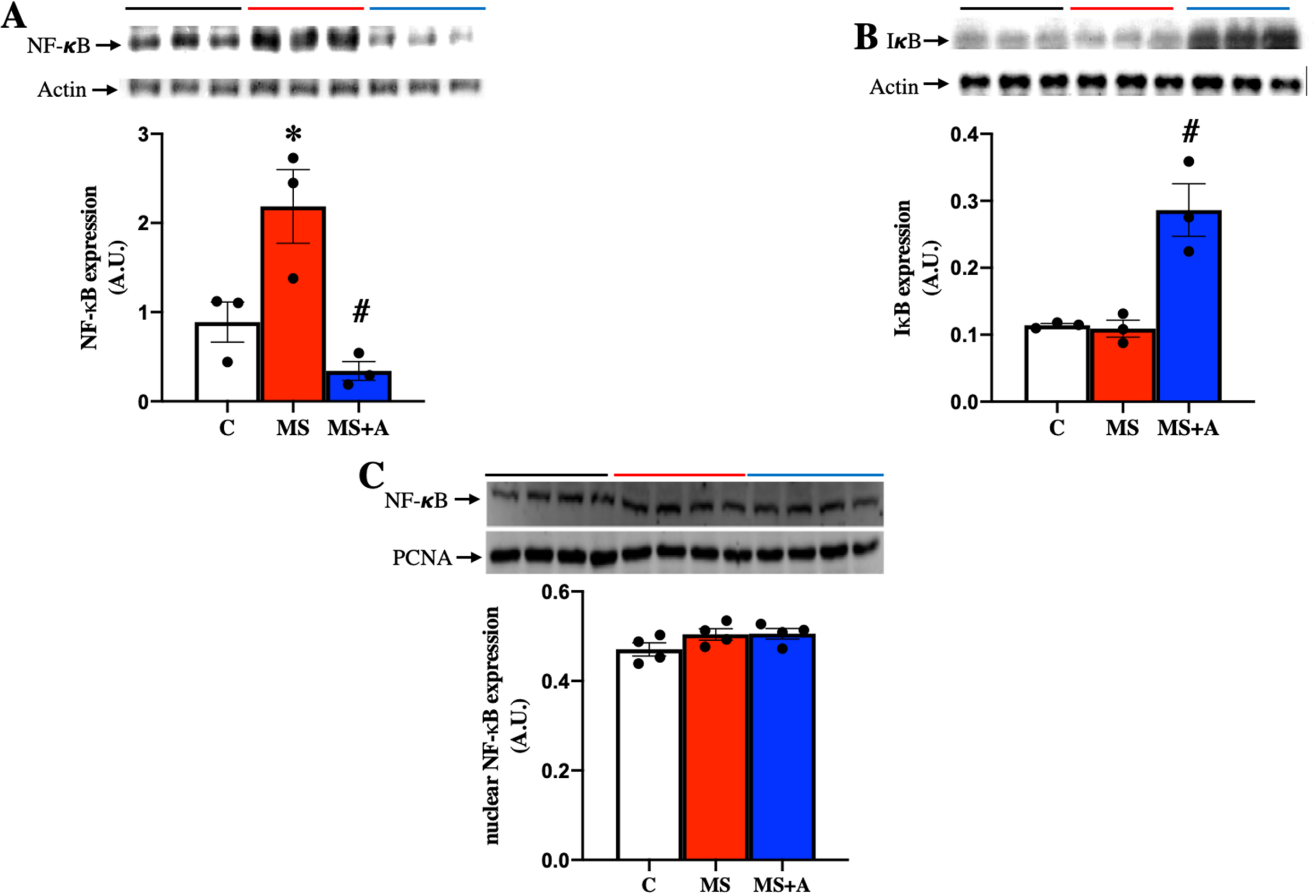
Effects of allicin on the expression of total nuclear factor kappa beta and its inhibitor. Total nuclear factor kappa beta (A) and its inhibitor (IκB) (B) in the kidney cortex, and NF-κB expression in nuclear extracts of kidney cortex (C) at 60 days of Follow-up. For representative Western blotting, three randomly selected samples per group were analysed respectively. C, control; MS, metabolic syndrome; MS+A. Data are expressed as mean ± SEM and analysed by one-way ANOVA. Statistical significance was established as *p < 0.05 *vs.* C; ^#^*p* < 0.05 *vs.* MS.

To explore the possible mechanism involved in the inflammatory response at the renal level during MS, the protein expression of nuclear factor-κB (NF-κB) and its inhibitor (IκB) were evaluated in renal cortex homogenates. MS induced the overexpression of NF-κB compared to the C group, which the allicin treatment prevented (Fig. 7A). IκB was not different between Control and MS groups; however, allicin treatment increased it by 2.5-fold its expression (Fig. 7B). NF-κB has to be translocated to the nucleus to induce the expression of proinflammatory cytokines, thus, we evaluated the nuclear expression of NF-κB. We did not observe statistical differences in the nuclear expression of NF-κB in the three experimental groups. However, there was a tendency to increase its expression in the MS and MS+A groups when compared with the C group (Fig. 7C).

## Discussion

MS includes abdominal obesity, hyperglycemia, dyslipidemia, hypertension, oxidative stress, and inflammation. Several reports describe that each one of the components of the MS can lead to CKD, but the more components there are, the greater the risk of suffering from CKD (Huh et al., 2017; Kawamoto et al., 2019; Wu et al., 2021; Alizadeh et al., 2018). Previous studies have reported beneficial effects of allicin including antioxidant, anti-inflammatory, antidiabetic, antihypertensive, cardioprotective, and nephroprotective, which suggest that it could be helpful in the control of risk factors in MS and, thus, preventing the development and progression of CKD. Allicin has been administered simultaneous at the time of injury induction or before the establishment of the diseases, so allicin acted as a preventive. This research demonstrated the kidney disease caused by MS before allicin treatment started. This methodological strategy was carried out to resemble what happens in the clinic setting, meaning the treatment prescription after the disease induction.

Allicin fully reversed the weight gain in this study, lowered serum glucose, TC, and LDL-c levels, and increased HDL-c levels. Other studies have reported that allicin reduced weight gain, visceral and subcutaneous fat accumulation, and improved OGTT and dyslipidemia. The effects on lipids were through increased expression of genes involved in lipolysis (hormone-sensitive lipase (HSL), adipose triglyceride lipase (ATGL) and lipoprotein lipase (LPL)), and in the signaling pathway of insulin (insulin receptor substrate 1 (IRS-1) and IRS-2). Also, allicin downregulated the expression of lipogenic genes (SREBP1, acetyl-CoA carboxylase (ACC), fatty acid synthase (FASN), stearoyl-CoA desaturase-1 (SCD-1), and activated receptor by peroxisome proliferator-γ (PPARγ)) (Shi et al., 2019; Ashraf et al., 2005; Cannizzaro et al., 2017); this is particularly important as up-regulation of fatty acid synthesis is closely related with mitochondrial bioenergetics impairment in various models or renal damage, such as the induced by the unilateral ureteral obstruction, folic acid, and nephrectomy (Ceja-Galicia et al., 2022; Martínez-Klimova et al., 2020; Kang et al., 2015). Furthermore, our results showed that allicin improved the electron flow in the mitochondria suggesting that restoration of mitochondrial bioenergetics could lead to protective effects in renal and plasma lipids. However more profound studies are still necessary to clarify such effects.

Allicin showed an antihypertensive effect in this MS model. This data agree with other studies using different experimental models of hypertension (García-Trejo et al., 2017; Cui et al., 2020). It has been suggested that the antihypertensive and vasodilative effects of allicin are mediated by its effects on the endothelium, specifically on the nitric oxide-soluble guanylyl cyclase-cyclic guanosine monophosphate (NO-sGC-cGMP), prostaglandin-adenylyl cyclase-cyclic adenosine monophosphate (PGI_2_-AC-cAMP), and endothelium-derived hyperpolarizing factor (EDHF) pathways. Also, in a model of CKD, allicin exerted antihypertensive effects through the downregulation of angiotensin II receptor type 1 (AT1R) and upregulation of angiotensin II receptor type II (ATR2) (García-Trejo et al., 2016, 2017; Cui et al., 2020).

During MS, there are changes in renal function, such as proteinuria, microalbuminuria, and increased plasma creatinine levels, which correlate with structural damage at glomerular and tubular levels (Huh et al., 2017; García-Arroyo et al., 2021; Markova et al., 2019; Kittiskulnam et al., 2018; Sun et al., 2020; Szeto et al., 2016). We observed that allicin treatment decreased proteinuria, microalbuminuria, plasma creatinine levels, and glomerular and tubular damage markers (nephrin, KIM-1, and NGAL). These results confirm that allicin has nephroprotective effects and demonstrated that controlling the MS risk factors contributes to protecting kidney.

On the other hand, the adipose tissue stores energy as triglycerides, and secrete hormones and inflammatory mediators such as IL-1β, IL-6, and TNF-α, and hosts macrophages, which are the majority population of leukocytes in the expansion of adipose tissue (Monserrat-Mesquida et al., 2020; Quetglas-Llabrés et al., 2022). In experimental MS, it was observed that infiltration of macrophages in perirenal tissue could be a pathway by which proinflammatory cytokines access into the kidney, excessive production of proinflammatory cytokines induces renal dysfunction and insulin resistance, which in turn leads to sodium retention, vasoconstriction, and activation of the RAAS, resulting in glomerular hyperfiltration, glomerulomegaly, and segmental sclerosis (Lin et al., 2022).

Here, we confirmed that MS induces a proinflammatory state by overexpressing IL-1β, IL-6, and TNF-α at systemic and renal levels, which was reversed by allicin treatment. This anti-inflammatory effect likely impacted kidney function positively. *In vivo* and *in vitro* allicin regulates the expression of IL-1β, IL-6 and TNF-α, both at the mRNA and protein levels (Qian et al., 2018; Alam et al., 2018). Other studies reported that allicin attenuates the inflammatory response in the kidney and cardiomyocytes in cell culture under experimental conditions of ischemia-reperfusion and hypoxia-reoxygenation respectively (Shan et al., 2021; Deng et al., 2021).

NF-κB signaling pathway has been extensively studied in MS and is responsible for producing proinflammatory cytokines (Catrysse & van Loo, 2017; Benzler et al., 2015). The activation of NF-κB in the cytoplasm depends mainly on the phosphorylation of the inhibitory protein IκB, which is subsequently degraded by the proteasome. Consequently, NF-κB migrates to the nucleus, leading to the expression of proinflammatory cytokines. In the present study, cytoplasmic NF-κB expression was increased in the renal cortex of rats with MS, and allicin decreased it, while the expression of IκB was not different between MS and control groups; however, allicin increased the expression of this protein. The nuclear translocation of NF-κB protein expression showed a trend to increase in the MS and allicin did not revert this tendency, despite a significant reduction in inflammation markers were observed. This effect might possibly due to the stimulation of posttranslational mechanisms that prevent the expression of inflammatory mediators. On the other hand, some studies have shown that the anti-inflammatory effects of allicin were mediated by an increase in the expression of IκB (Arellano-Buendía et al., 2018; Benzler et al., 2015; Sánchez-Gloria et al., 2021).

On the other hand, the modulation of inflammation also may be induced through the crosstalk between Nrf2-NF-κB pathways (Ahmed et al., 2017). In this context, it has been described that allicin reduced inflammation caused by diabetic macroangiopathy in mice through the Nrf2-NF-κB pathway and improved osteoarthritis by downregulating PI3K/Akt/NF-κB signaling (Li et al., 2020; Qian et al., 2018). Therefore, it is possible that the anti-inflammatory effects of allicin observed in our study are related to the Nrf2 pathway, and further studies are needed to explore this mechanism.

Another mechanism involved in kidney damage caused by MS is oxidative stress. Excessive production of reactive oxygen species (ROS) and nitrogen (RNS) react and oxidize lipids, proteins, and nucleic acids (Monserrat-Mesquida et al., 2020; Quetglas-Llabrés et al., 2022). Infiltration of inflammatory cells in the kidney produces ROS and damages proximal tubules by interfering with the tubular transport of ions altering renal hemodynamics (Lin et al., 2022). Our results showed an increase in the levels of oxidative stress markers in the MS group that were attenuated with allicin. This is in agreement with our results that showed mitochondrial bioenergetics alteratons. In fact, mitochondria are one of the main sites of ROS production (Brillo et al., 2021). Furthermore, previous studies showed that the decrement in CI activity increased hydrogen peroxide production by renal mitochondria, resulting in higher oxidative stress promoting fibrosis and inflammation in experimental CKD (Aparicio-Trejo et al., 2020a, 2020b; Jiménez-Uribe et al., 2021), suggesting that allicin protects mitochondria by preventing the increase in ROS production.

On the other hand, it has been reported that allicin increases intracellular levels of glutathione, which may be related to its antioxidant properties. This agrees with the increase in GPx activity observed in MS+A group. Also, it has been described that allicin readily undergoes a Cope elimination reaction at room temperature to form allylsulfenic acid, which can act as antioxidant, but, in chemical synthesis two molecules of allylsulfenic acid can be converted back to allicin (Zhou et al., 2022). Furthermore, allicin could induce mild oxidative stress in cells, activating hormeic responses and making cells more resistant to further oxidative stress damage (Borlinghaus et al., 2021).

In the cells there are endogenous antioxidants modulated by the NF-E2-related factor 2 (Nrf2)-Kelch-like ECH-associated protein 1 (Keap1) (Nrf2/Keap1) master regulator. Under basal conditions, Nrf2 is sequestered by cytoplasmic Keap1 and targeted for proteasomal degradation; but when intracellular oxidative stress increases, Nrf2 detaches from Keap1 and translocates to the nucleus, where it heterodimerizes with one of the small musculoaponeurotic fibrosarcoma oncogene homolog (Maf) proteins. The heterodimers recognize the antioxidant response element (AREs) enhancer sequences in the regulatory regions of the Nrf2 target genes that encode antioxidant and detoxifying molecules (Bellezza et al., 2018). In our study, we observed decreased nuclear translocation of Nrf2 in MS, which was alleviated with the allicin treatment. Other studies, report that allicin decreased liver damage by activating the Nrf2/ARE pathway (Sun et al., 2021). Previously, in experimental models of CKD by renal ablation and diabetes, we observed an antioxidant effect of allicin by a mechanism dependent on Nrf2/Keap1 pathway (García-Trejo et al., 2016; Arellano-Buendía et al., 2018, 2020). Additionally, we observed an increase in SOD and GPx activities with the allicin treatment, suggesting that allicin can activate this protective pathway in the kidney.

## Conclusions

Allicin exerts its beneficial effects on metabolic syndrome by significantly reducing systemic and renal inflammation and oxidative stress. Part of these effects were likely mediated through the Nrf2-NF-κB pathway crosstalk in renal tissue. Moreover, allicin also reversed bioenergetics alterations; however, more profound mitochondrial studies are still necessary. These results suggest allicin may be a therapeutic alternative for treating kidney injury induced by metabolic syndrome risk factors. Because metabolic syndrome mainly affects the most disadvantaged populations, finding accessible and inexpensive treatment alternatives is urgently needed.

## Supplemental Information

10.7717/peerj.16132/supp-1 Supplemental Information 1 Raw data. Each table indicates the value of one determination

10.7717/peerj.16132/supp-2 Supplemental Information 2 Uncropped blots. Each blot represents the three experimental groups.

10.7717/peerj.16132/supp-3 Supplemental Information 3 Author checklist.

10.7717/peerj.16132/supp-4 Supplemental Information 4 Activity of mitochondrial complexes I and II in cortex at 30 days. C: control and MS: metabolic syndrome. Data are expressed as mean ± SEM, analysed by unpaired T test with Welch’s correction.

10.7717/peerj.16132/supp-5 Supplemental Information 5 Effects of allicin on the enzymatic activities of superoxide dismutase (SOD) (A) and glutathione peroxidase (Gpx) (B) in renal cortex at 60 days of follow-up. C: control; MS: metabolic syndrome and MS+A: metabolic syndrome + allicin. Data are expressed as mean ± SEM, and analysed by one-way ANOVA.

## Additional Information and Declarations

### Data Availability

The following information was supplied regarding data availability: The western blot of experiments are available in the Supplemental File.
