# Supplementary material for: Antioxidant and anti-inflammatory effects of allicin in the kidney of an experimental model of metabolic syndrome
Source: PeerJ. 2023 Sep 27;11:e16132. doi: 10.7717/peerj.16132 (PMC10541809; doi:10.7717/peerj.16132)
Supplement: Supplemental Information 1 — Each table indicates the value of one determination [file peerj-11-16132-s001.docx]

Results 30 days

Body weight

| Control | MS |
| --- | --- |
| 36 | 75 |
| 52 | 125 |
| 19 | 79 |
| 20 | 121 |
| 82 | 127 |
| 34 | 98 |

Systolic blood pressure

| Control | MS |
| --- | --- |
| 113.50 | 122 |
| 113.33 | 126 |
| 114.60 | 135.75 |
| 107.33 | 145.50 |
| 111.50 | 139.60 |
| 114.50 | 130.60 |

Blood glucose

| Control | MS |
| --- | --- |
| 78 | 110 |
| 77 | 100 |
| 90 | 110 |
| 76 | 126 |
| 80 | 117 |
| 89 | 123 |

Plasma Triglycerides

| Control | MS |
| --- | --- |
| 43.1372549 | 117.64705990 |
| 31.3725490 | 223.5294120 |
| 62.7450980 | 247.0588240 |
| 31.3725490 | 192.1568630 |
| 15.6862745 | 62.7450980 |
| 53.6373187 | 125.6734529 |

Total Cholesterol

| Control | MS |
| --- | --- |
| 67.30 | 70.5 |
| 66.40 | 131.0 |
| 50.00 | 141.9 |
| 81.40 | 117.8 |
| 65.00 | 79.1 |
| 58.98 | 119.8 |

Plasma LDL-c

| Control | MS |
| --- | --- |
| 13.393 | 48.548 |
| 11.719 | 48.343 |
| 21.763 | 36.830 |
| 21.783 | 40.178 |
| 6.696 | 40.200 |
| 6.703 | 51.896 |

Plasma HDL-c

| Control | MS |
| --- | --- |
| 19.70 | 3.5 |
| 24.30 | 8.9 |
| 13.90 | 4.3 |
| 36.00 | 3.0 |
| 22.60 | 9.3 |
| 16.43 | 4.7 |

Diuresis

| Control | MS |
| --- | --- |
| 17 | 30 |
| 12 | 43 |
| 11 | 70 |
| 9 | 65 |
| 20 | 40 |
| 15 | 60 |

Proteinuria

| Control | MS |
| --- | --- |
| 19.2800000 | 44.9175 |
| 15.8410000 | 34.18500 |
| 20.2290000 | 34.99125 |
| 7.83515500 | 30.27375 |
| 4.60000000 | 35.88000 |
| 10.7782349 | 34.58000 |

Plasma creatinine

| Control | MS |
| --- | --- |
| 0.17391304 | 0.60869565 |
| 0.17351302 | 0.43478261 |
| 0.34782609 | 0.69565217 |
| 0.17405223 | 0.78260870 |
| 0.18356420 | 0.69565217 |
| 0.19653700 | 0.43478261 |

Mitochondrial respiratory complex 1 activity

| Control | MS |
| --- | --- |
| 45.368 | 41.261 |
| 67.334 | 38.321 |
| 73.300 | 40.038 |
| 78.058 | 43.354 |
| 76.541 | 41.669 |
| 82.278 | 25.499 |

Mitochondrial respiratory complex 2 activity

| Control | MS |
| --- | --- |
| 24.765 | 9.216 |
| 23.923 | 10.266 |
| 39.683 | 14.580 |
| 25.276 | 16.092 |
| 34.798 | 20.450 |
| 32.692 | 12.372 |

Results 60 days

Body weight

| Control | MS | MS+A |
| --- | --- | --- |
| 42 | 75 | 38 |
| 34 | 42 | 19 |
| 57 | 65 | 50 |
| 44 | 47 | 40 |
| 31 | 85 | 29 |
| 36 | 51 | 14 |

Systolic blood pressure

| Control | MS | MS+A |
| --- | --- | --- |
| 116.66 | 163.0 | 135.75 |
| 119.00 | 136.0 | 113.00 |
| 115.00 | 135.0 | 128.66 |
| 111.00 | 157.0 | 144.00 |
| 117.33 | 148.3 | 127.60 |
| 102.00 | 138.6 | 133.25 |

Blood glucose

| Control | MS | MS+A |
| --- | --- | --- |
| 116.66 | 163.0 | 135.75 |
| 119.00 | 136.0 | 113.00 |
| 115.00 | 135.0 | 128.66 |
| 111.00 | 157.0 | 144.00 |
| 117.33 | 148.3 | 127.60 |
| 102.00 | 138.6 | 133.25 |

Plasma Triglycerides

| Control | MS | MS+A |
| --- | --- | --- |
| 100.1126270 | 153.2974600 | 137.6548620 |
| 53.18483290 | 106.3696660 | 84.4700288 |
| 81.3415092 | 90.7270680 | 109.4981850 |
| 103.241146 | 106.3596670 | 59.4418721 |
| 85.7376554 | 150.1689400 | 78.2129896 |
| 81.3414089 | 140.7833810 | 81.3415092 |

Total Cholesterol

| Control | MS | MS+A |
| --- | --- | --- |
| 64.6 | 137.30 | 102.8 |
| 48.7 | 98.70 | 93.2 |
| 54.6 | 134.16 | 116.0 |
| 64.6 | 131.40 | 120.5 |
| 66.4 | 146.90 | 69.6 |
| 59.5 | 125.34 | 92.3 |

Plasma LDL-c

| Control | MS | MS+A |
| --- | --- | --- |
| 15.067 | 97.096 | 61.941 |
| 8.370 | 71.985 | 48.548 |
| 21.763 | 105.467 | 31.807 |
| 11.719 | 93.748 | 61.941 |
| 11.718 | 92.074 | 61.898 |
| 14.230 | 87.828 | 50.222 |

Plasma HDL-c

| Control | MS | MS+A |
| --- | --- | --- |
| 15.1 | 8.0 | 22.60 |
| 14.3 | 6.0 | 18.50 |
| 14.7 | 9.3 | 12.60 |
| 19.3 | 8.9 | 20.55 |
| 19.7 | 10.1 | 11.80 |
| 16.5 | 7.8 | 13.50 |

Diuresis

| Control | MS | MS+A |
| --- | --- | --- |
| 15.0 | 41.0 | 41.0 |
| 23.0 | 25.0 | 16.0 |
| 22.0 | 46.0 | 14.0 |
| 21.0 | 38.0 | 18.0 |
| 20.0 | 61.0 | 31.0 |
| 14.0 | 49.0 | 18.0 |

Proteinuria

| Control | MS | MS+A |
| --- | --- | --- |
| 21.640000 | 29.61589 | 15.87250 |
| 5.400500 | 37.20625 | 8.54625 |
| 17.143500 | 65.27400 | 18.76700 |
| 4.947125 | 23.01000 | 13.89150 |
| 7.203000 | 22.24975 | 8.53200 |
| 8.223112 | 34.75325 | 12.67452 |

Microalbuminuria

| Control | MS | MS+A |
| --- | --- | --- |
| 0.475 | 0.62 | 0.820 |
| 0.460 | 1.25 | 0.620 |
| 0.700 | 0.75 | 0.250 |
| 0.583 | 2.45 | 0.280 |
| 0.633 | 1.75 | 0.850 |
| 0.487 | 2.25 | 0.653 |

Plasma creatinine

| Control | MS | MS+A |
| --- | --- | --- |
| 0.13 | 0.53 | 0.47 |
| 0.20 | 0.53 | 0.33 |
| 0.40 | 0.73 | 0.47 |
| 0.20 | 0.80 | 0.40 |
| 0.13 | 0.80 | 0.60 |
| 0.27 | 0.67 | 0.36 |

4-HNE

| Control | MS | MS+A |
| --- | --- | --- |
| 1.845623 | 3.234650 | 2.567120 |
| 1.569860 | 3.456320 | 2.347650 |
| 1.648754 | 3.118750 | 2.229870 |
| 1.589860 | 3.376500 | 2.449870 |
| 1.756720 | 3.119870 | 2.498733 |
| 1.697650 | 3.986744 | 2.438763 |

DNPH

| Control | MS | MS+A |
| --- | --- | --- |
| 0.00183238 | 0.00351601 | 0.00180293 |
| 0.00195217 | 0.00327869 | 0.00156612 |
| 0.00100773 | 0.00312625 | 0.00133223 |
| 0.00012925 | 0.00305792 | 0.00214688 |
| 0.00216706 | 0.00311827 | 0.00187299 |
| 0.00153980 | 0.00201560 | 0.00201560 |

Antioxidant activity of SOD

| Control | MS | MS+A |
| --- | --- | --- |
| 31.118 | 25.027 | 42.478 |
| 35.242 | 20.909 | 35.588 |
| 49.029 | 29.471 | 31.145 |
| 38.421 | 21.608 | 39.692 |
| 51.092 | 25.125 | 37.560 |
| 55.200 | 24.463 | 34.224 |

Antioxidant activity of GPx

| Control | MS | MS+A |
| --- | --- | --- |
| 0.021 | 0.018 | 0.036 |
| 0.026 | 0.016 | 0.023 |
| 0.026 | 0.020 | 0.028 |
| 0.043 | 0.014 | 0.024 |
| 0.034 | 0.015 | 0.026 |
| 0.029 | 0.016 | 0.024 |

Mitochondrial respiratory complex 1 activity

| Control | MS | MS+A |
| --- | --- | --- |
| 45.368 | 46.3500 | 78.964 |
| 77.434 | 24.4920 | 48.906 |
| 77.300 | 39.8250 | 50.443 |
| 68.078 | 24.0260 | 66.795 |
| 86.145 | 42.6990 | 49.791 |
| 86.324 | 27.0782 | 51.305 |

Mitochondrial respiratory complex 2 activity

| Control | MS | MS+A |
| --- | --- | --- |
| 26.553 | 10.984 | 21.451 |
| 33.434 | 11.314 | 27.286 |
| 19.683 | 9.782 | 28.707 |
| 25.144 | 14.566 | 27.406 |
| 39.256 | 7.856 | 21.377 |
| 22.890 | 13.896 | 19.236 |

RESULTS WESTERN BLOT 60 days

Nephrin

| Control | MS | MS+A |
| --- | --- | --- |
| 0.26756826 | 0.58693137 | 0.44730379 |
| 0.31435903 | 0.7559204 | 0.40006539 |
| 0.38449832 | 0.61326387 | 0.31221164 |

KIM-1

| Control | MS | MS+A |
| --- | --- | --- |
| 0.26095413 | 1.54588034 | 1.50730137 |
| 0.42652859 | 2.32291667 | 1.45934071 |
| 0.30817954 | 1.95517039 | 1.24117737 |

NGAL

| Control | MS | MS+A |
| --- | --- | --- |
| 0 | 11.9233125 | 7.101675 |
| 0 | 13.123575 | 6.30837 |
| 0.40128 | 11.0952 | 6.75189 |

NRF2

| Control | MS | MS+A |
| --- | --- | --- |
| 0.9036351 | 0.16613908 | 1.02033784 |
| 0.7386571 | 0.25001137 | 0.91598617 |
| 0.4927687 | 0.36105074 | 0.61511771 |

Nuclear Nrf2

| Control | MS | MS+A |
| --- | --- | --- |
| 0.7813 | 0.4892 | 0.7328 |
| 0.7522 | 0.5318 | 0.7527 |
| 0.7229 | 0.5522 | 0.7218 |
| 0.7712 | 0.5036 | 0.7715 |

Keap-1

| Control | MS | MS+A |
| --- | --- | --- |
| 0.80849071 | 1.5128852 | 1.01178448 |
| 1.21660549 | 1.5712122 | 0.90717417 |
| 0.70757267 | 1.55824824 | 0.47600252 |

IL-1β serum

| Control | MS | MS+A |
| --- | --- | --- |
| 58.57656 | 109.550933 | 42.566888 |
| 57.871744 | 96.164656 | 47.339488 |
| 67.70232 | 106.099243 | 56.1993413 |

IL-6 serum

| Control | MS | MS+A |
| --- | --- | --- |
| 73.934224 | 150.927019 | 94.1152333 |
| 83.7879467 | 163.850717 | 87.799712 |
| 79.11904 | 143.409933 | 74.699716 |

IL-1β cortex

| Control | MS | MS+A |
| --- | --- | --- |
| 0.1778657 | 0.57730302 | 0.18962919 |
| 0.12681788 | 0.46165794 | 0.10364365 |
| 0.2437578 | 0.37940105 | 0.25062966 |

IL-6 cortex

| Control | MS | MS+A |
| --- | --- | --- |
| 0.07426462 | 0.455284 | 0.17485132 |
| 0.11363926 | 0.47118074 | 0.18849277 |
| 0.21890359 | 0.27816807 | 0.21761979 |

TNF-α

| Control | MS | MS+A |
| --- | --- | --- |
| 0.00548684 | 0.05248923 | 0.01065938 |
| 0.00978655 | 0.05246586 | 0.009241 |
| 0.01319059 | 0.02187773 | 0.01937812 |

NFκ-B

| Control | MS | MS+A |
| --- | --- | --- |
| 0.4407904 | 2.44968786 | 0.54146943 |
| 1.10462634 | 2.72990143 | 0.29019351 |
| 1.121796 | 1.37925282 | 0.18903005 |

Nuclear NFκ-B

| Control | MS | MS+A |
| --- | --- | --- |
| 0.4875 | 0.4763 | 0.5274 |
| 0.4529 | 0.5126 | 0.5086 |
| 0.4389 | 0.5346 | 0.5139 |
| 0.5029 | 0.4926 | 0.4722 |

IκB

| Control | MS | MS+A |
| --- | --- | --- |
| 0.11809664 | 0.08792671 | 0.2244872 |
| 0.11496125 | 0.10817188 | 0.27545489 |
| 0.10970363 | 0.13151575 | 0.3589636 |
