# Supplementary figures and images for: Antioxidant and anti-inflammatory effects of allicin in the kidney of an experimental model of metabolic syndrome
Source: PeerJ. 2023 Sep 27;11:e16132. doi: 10.7717/peerj.16132 (PMC10541809; doi:10.7717/peerj.16132)

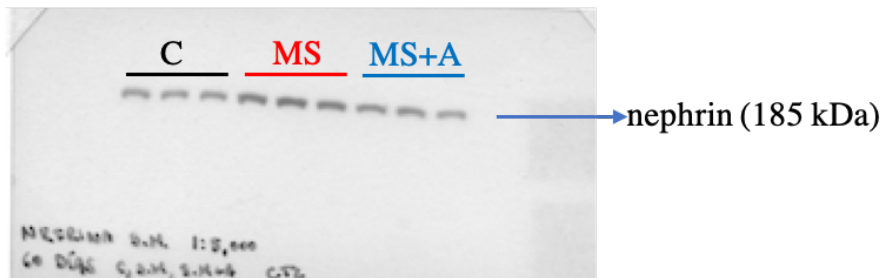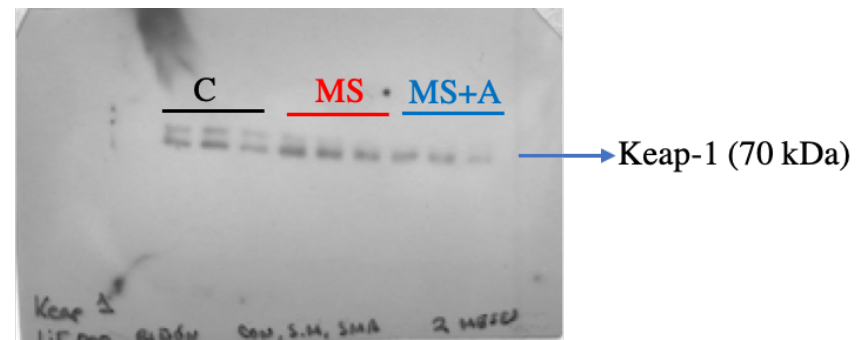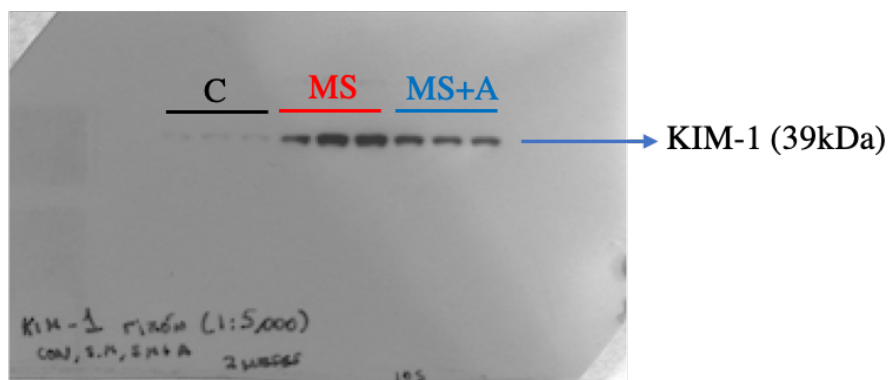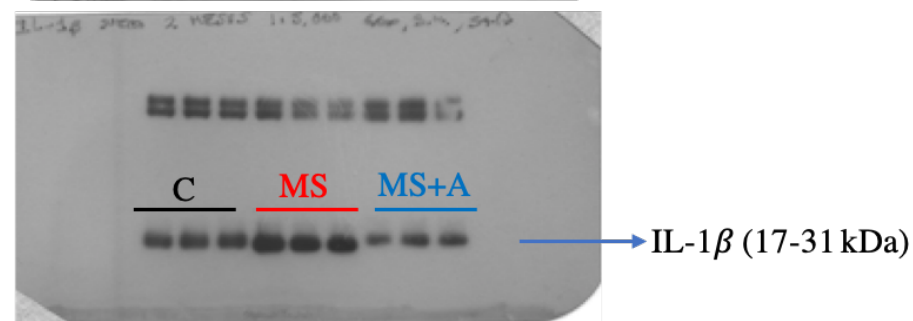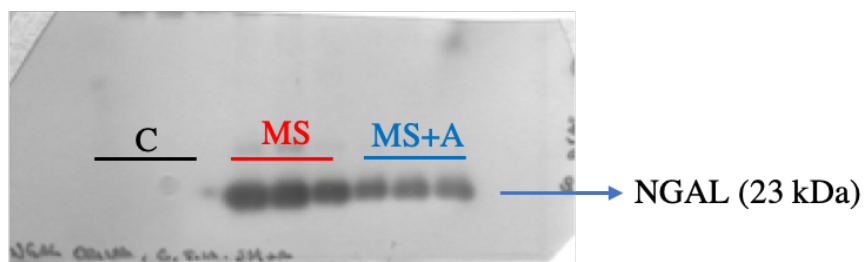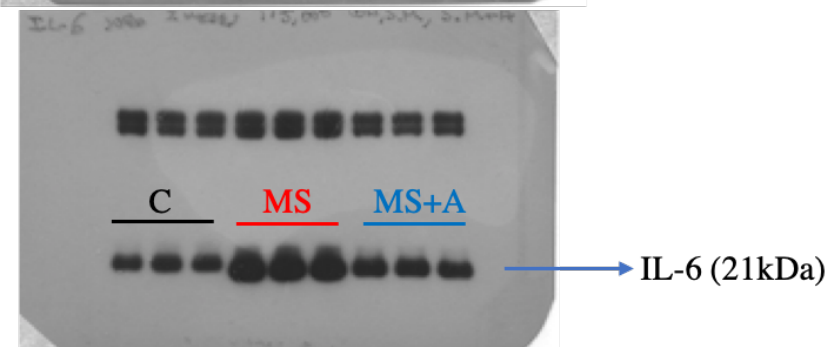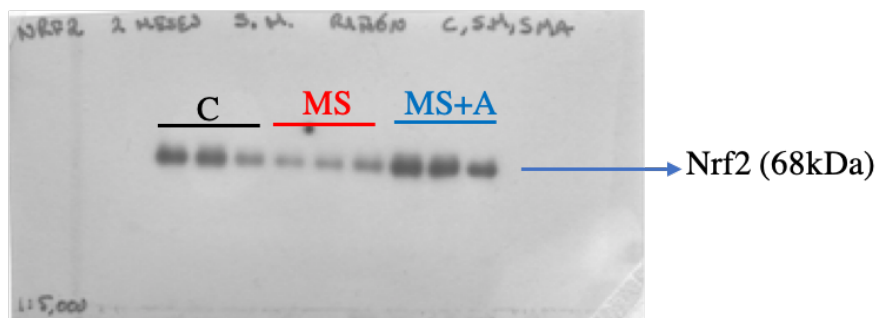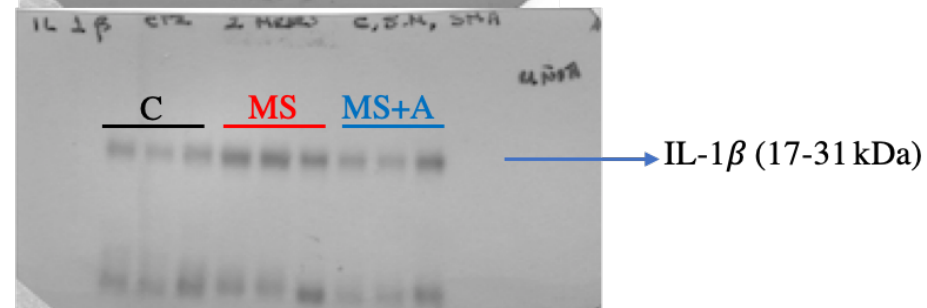

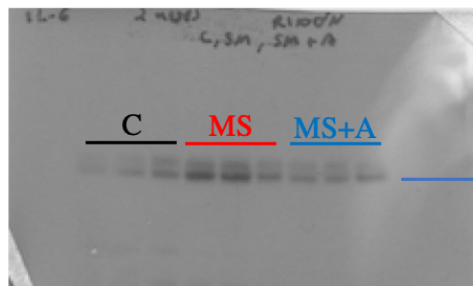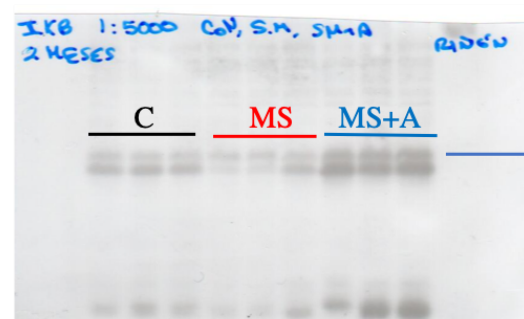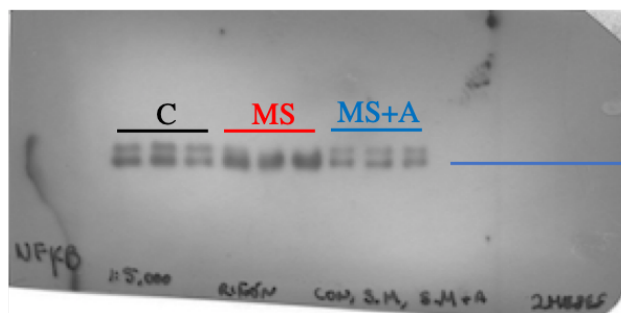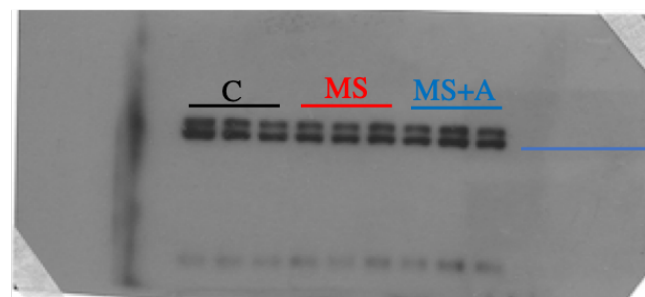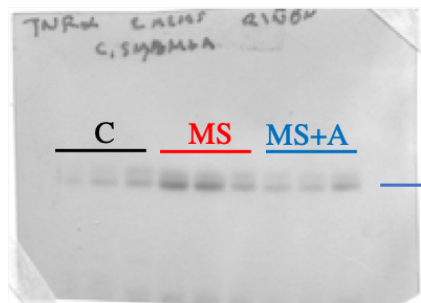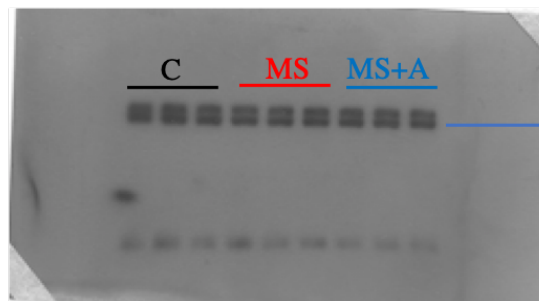

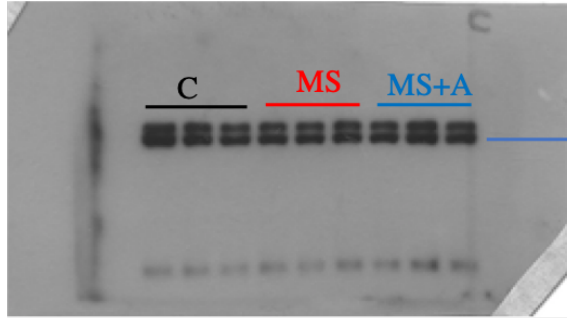

Actin (43kDa)

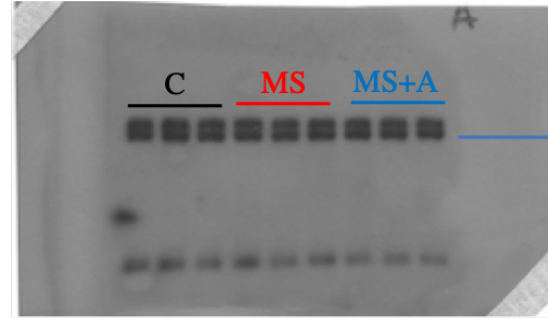

Actin (43kDa)

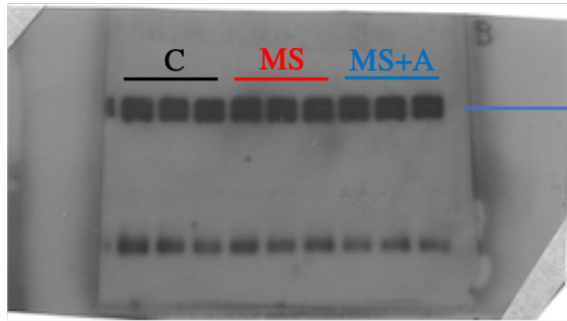

Actin (43kDa)

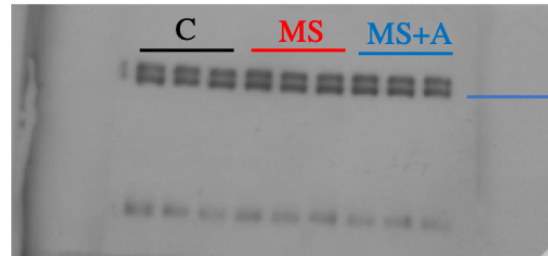

Actin (43kDa)

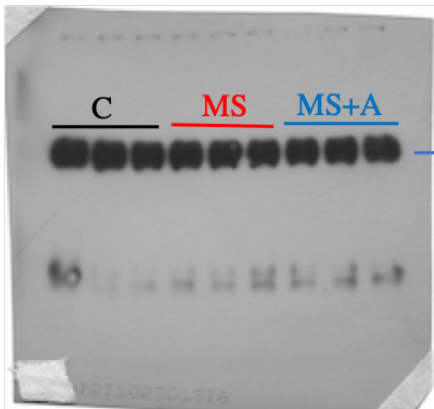

Actin (43kDa)

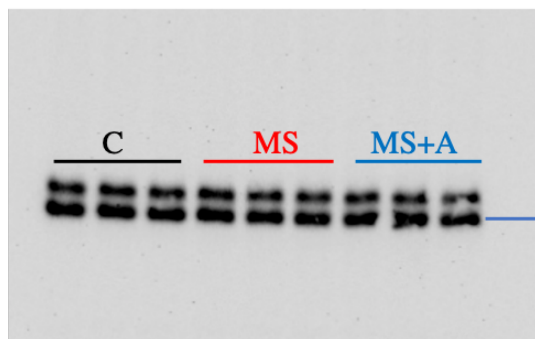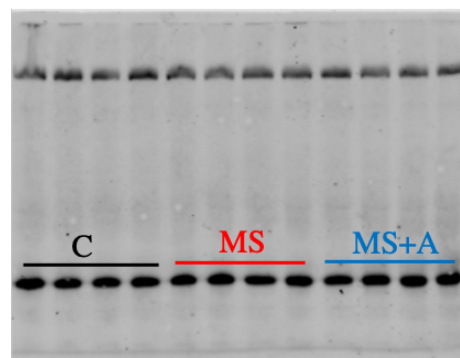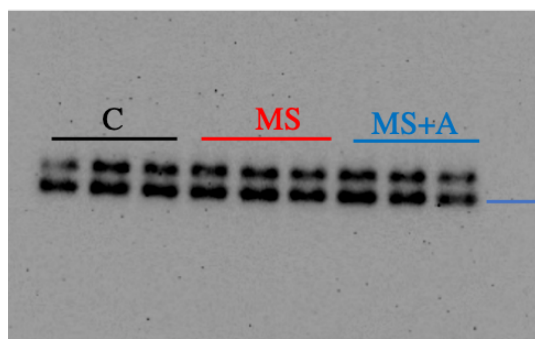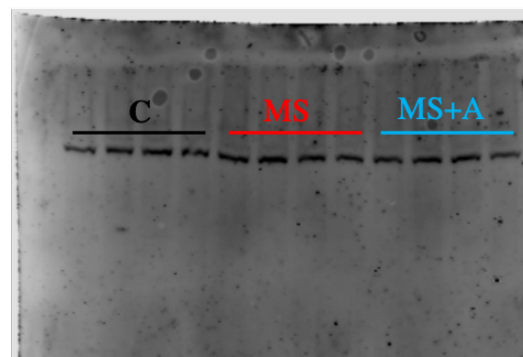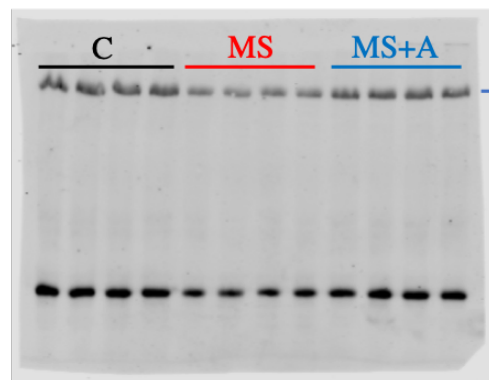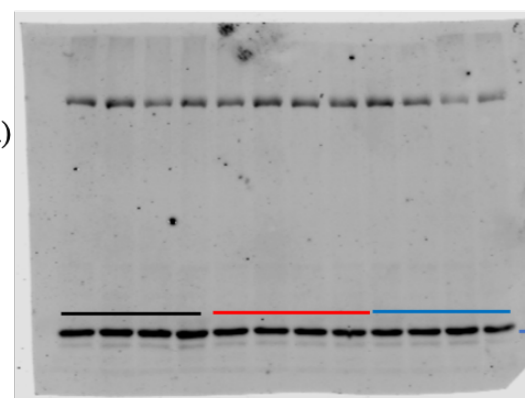

Supplement: Supplemental Information 2 — Each blot represents the three experimental groups. [file peerj-11-16132-s002.pdf]

# Complex I

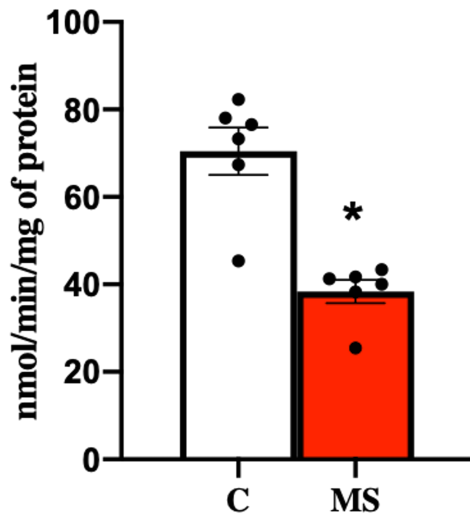

# Complex II

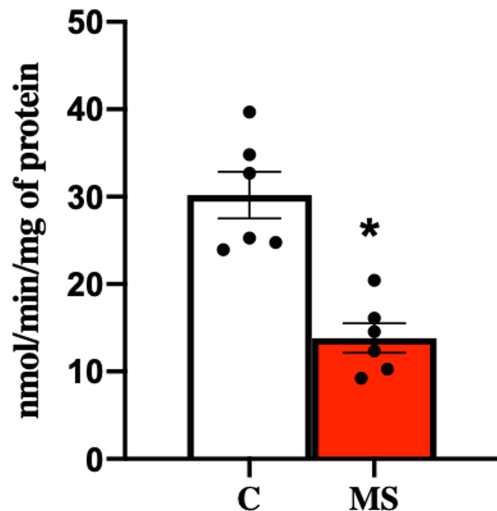

Supplement: Supplemental Information 4 — C: control and MS: metabolic syndrome. Data are expressed as mean ± SEM, analysed by unpaired T test with Welch’s correction. [file peerj-11-16132-s004.pdf]

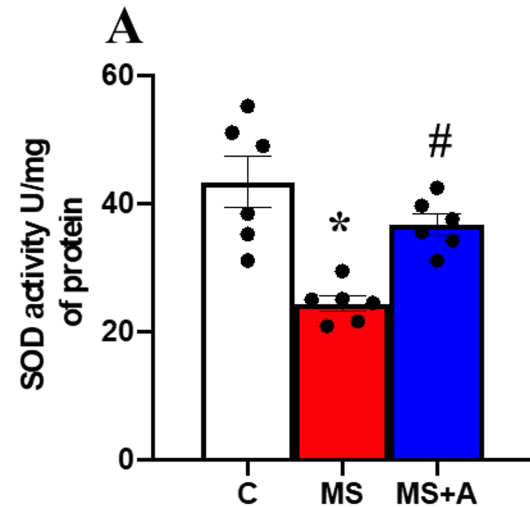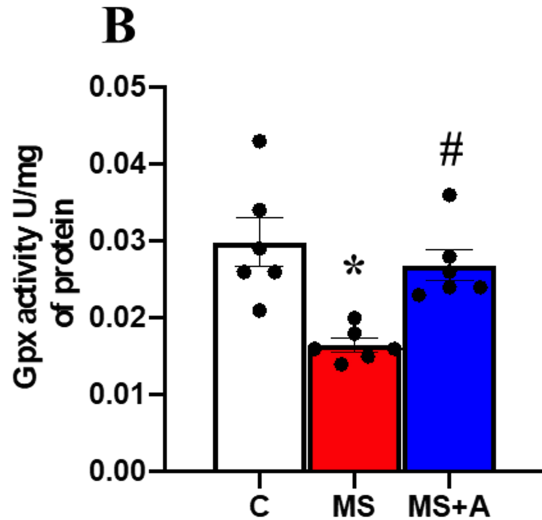

Supplement: Supplemental Information 5 — C: control; MS: metabolic syndrome and MS+A: metabolic syndrome + allicin. Data are expressed as mean ± SEM, and analysed by one-way ANOVA. [file peerj-11-16132-s005.pdf]
